# Wiskott–Aldrich Syndrome: Immunodeficiency resulting from defective cell migration and impaired immunostimulatory activation

**DOI:** 10.1016/j.imbio.2009.06.009

**Published:** 2009-09

**Authors:** Gerben Bouma, Siobhan O. Burns, Adrian J. Thrasher

**Affiliations:** a Centre for Immunodeficiency, Molecular Immunology, UCL Institute of Child Health, Molecular Immunology, 30 Guilford Street, London WC1N 1EH, UK; b Great Ormond Street Hospital for Children NHS Trust, Great Ormond Street, London, UK

**Keywords:** WAS, Wiskott–Aldrich Syndrome, WASp, WAS protein, DC, dendritic cell, XLT, X-linked neutropenia, WAS KO, WASp knockout, VCA, verprolin homology, central and acidic, GBD, GTPase binding domain, TCR, T cell receptor, IS, immunological synapse, SMAC, supramolecular activation cluster, Wiskott–Aldrich Syndrome, WASp, Dendritic cells, Migration, T lymphocytes, Immunological synapse, Cell activation

## Abstract

Regulation of the actin cytoskeleton is crucial for many aspects of correct and cooperative functioning of immune cells, such as migration, antigen uptake and cell activation. The Wiskott–Aldrich Syndrome protein (WASp) is an important regulator of actin cytoskeletal rearrangements and lack of this protein results in impaired immune function. This review discusses recent new insights of the role of WASp at molecular and cellular level and evaluates how WASp deficiency affects important immunological features and how defective immune cell function contributes to compromised host defence.

## Introduction

The actin cytoskeleton regulates many cellular functions that are related to the immune response, such as migration, extravasation, antigen uptake and recognition, and cell activation. An important regulator of the actin cytoskeleton is the Wiskott–Aldrich Syndrome protein (WASp) and patients who lack its expression have compromised immune function. Here we discuss at molecular and cellular level how WASp deficiency affects important immunological features, such as migration and cell activation, and how defective immune cell functioning contributes to a compromised host defence.

## Importance of the actin cytoskeleton for immune responses

Effective immunity is dependent on correct and cooperative function of all immune cell lineages. While the innate arm of the immune system is important in the first stages of infection, the adaptive arm is required to maintain long-term protection and memory. Both cells of the innate and of the adaptive arms require dynamic cytoskeletal rearrangements to allow effective cellular function. Recruitment of innate blood-borne neutrophils and monocytes to inflammatory sites is dependent on dynamic cell shape changes to allow the cells to diapedese from the blood stream, through the vascular endothelium into the underlying tissue (Vicente-Manzanares and Sanchez-Madrid 2004; Worthylake and Burridge 2001). Several steps are involved in this process of tethering, rolling and extravasation, and the actin cytoskeleton plays an important role in all of these individual steps. At the inflammatory site, phagocytosis of particulate antigens and uptake of soluble antigens through endocytosis/pinocytosis by neutrophils and macrophages requires protrusion and retraction of the plasma membrane, which is regulated by the actin cytoskeleton (Swanson 2008). Uptake of antigens by dendritic cells (DCs) in an inflammatory environment initiates maturation and migration of DC to the draining lymphoid tissues, where they present the captured antigen to T cells (Banchereau and Steinman 1998). Dynamic cytoskeletal rearrangement is mandatory for DC motility and for formation of the immunological synapse (IS) during T cell priming (Al-Alwan et al. 2001; Vicente-Manzanares and Sanchez-Madrid 2004). Primed T cells subsequently home to the inflammatory site to assist macrophages in the clearance of pathogens or to exert effector function such as killing of virus-infected cells by cytotoxic T cells. Again, for these processes a functional and dynamic cytoskeletal rearrangement machinery is essential (Vicente-Manzanares and Sanchez-Madrid 2004; Worthylake and Burridge 2001).

## Defective cytoskeletal rearrangements lead to Wiskott–Aldrich Syndrome

The Wiskott–Aldrich Syndrome protein is a key regulator of the actin cytoskeleton, transmitting and integrating actin-regulating signals that are essential for multiple cell functions, including cell motility and induction of cell shape change (Burns et al. 2004a; Thrasher 2002). Reduced or absent WASp expression may result in the primary immunodeficiency disorder Wiskott–Aldrich Syndrome (WAS), which is characterized by a classical triad of microthrombocytopenia, eczema and immune deficiency, affecting 1–10 in 1 000 000 live births (Ochs and Thrasher 2006).

The *WAS* gene is localised on the X chromosome and encodes the 502-amino-acid-long WASp. As WASp is exclusively expressed in haematopoietic cells, defective function has been described in most immune cell lineages, giving rise to a complex combined cellular and humoral immune deficiency, resulting in susceptibility to severe and life-threatening infections (Ochs and Thrasher 2006). Defective immunological function can include generalised lymphopenia, abnormal T cell proliferation (especially in response to CD3 stimulation) and aberrant immunoglobulin responses to protein and more particularly to polysaccharide antigens, including reduced isohaemagglutinin levels (Ochs et al. 1980; Sullivan et al. 1994; Park et al. 2004). In addition, failure of immunity may manifest as autoimmunity, which was seen in 40% and 72% of WAS patients in two independent studies (Dupuis-Girod et al. 2003; Sullivan et al. 1994). A wide range of autoimmune diseases can occur even as early as infancy, with autoimmune cytopenias, arthritis and vasculitis being the most common complications. While relatively understudied, recently new insights into the pathophysiology of WAS autoimmune features are emerging and several reports have shown defective suppressor function of regulatory T cells in WAS (Adriani et al. 2007; Humblet-Baron et al. 2007; Maillard et al. 2007; Marangoni et al. 2007). Clinically, WAS-related autoimmunity can be difficult to manage and is a poor prognostic indicator (Dupuis-Girod et al. 2003; Imai et al. 2004). Haematopoietic malignancies are an additional serious complication of WAS, which may result from defective immune surveillance, although the pathogenic mechanisms are presently unclear (Burns et al. 2004a; Shcherbina et al. 1999; Imai et al. 2004). Approximately 300 unique mutations in the *WAS* gene have been reported with five specific mutations occurring with high frequency (Jin et al. 2004; Notarangelo et al. 2005; Imai et al. 2003). Three of these mutations give rise to a milder clinical phenotype, which is mainly restricted to microthrombocytopenia and is referred to as X-linked thrombocytopenia (XLT; Jin et al. 2004; Ochs and Thrasher 2006). Intriguingly, mutations that give rise to constitutively active WASp result in a completely different clinical phenotype, characterised by congenital neutropenia and severe bacterial infections (Ancliff et al. 2006; Devriendt et al. 2001). Studies of the effects of WASp deficiency in vivo have been assisted by the generation of two separate WASp knockout (WAS KO) mouse models, both of which provide good mimics for the haematopoietic features of human WAS (Snapper et al. 1998; Zhang et al. 1999).

On the whole, XLT has a good prognosis with conservative treatment, although recent long-term studies suggest that the incidence of autoimmunity may be higher than previously thought (Imai et al. 2004). In contrast severely affected WAS patients have poor prognosis without treatment, with bleeding and severe infections constituting the major causes of morbidity and mortality in infancy and early childhood. The only curative therapy, at present, is bone marrow or haematopoietic stem cell transplantation. In the last decade good progress has been made in the development of corrective gene therapy for WAS. Many reports have shown that good expression of WASp and functional restoration of lymphocytes could be achieved, initially using gammaretroviral transdcution (Candotti et al. 1999; Dupre et al. 2004; Strom et al. 2003a; Wada et al. 2002) and more recently by lentiviral transduction (Charrier et al. 2007; Dupre et al. 2004; Martin et al. 2005). Furthermore, studies with WAS KO mice have demonstrated that gene therapy allows safe and long-term restoration of WASp expression and functionality in multiple immune cell lineages (Blundell et al. 2008; Charrier et al. 2005; Dupre et al. 2006; Frecha et al. 2008; Klein et al. 2003; Martin et al. 2005; Strom et al. 2003b). Gene therapy trials in human patients have recently been initiated (Notarangelo et al. 2005; Ochs and Thrasher 2006).

## Cytoskeletal rearrangements by WASp

WASp is a member of a family of proteins regulating cytoskeletal rearrangements. Other WASp family homologues are more widely expressed than WASp itself and in vertebrates these include ubiquitously expressed neural WASp (N-WASp) and three homologues of WASP family Verprolin-homologous protein (WAVE), also called suppressor of G-protein coupled cyclic-AMP receptor (SCAR; Takenawa and Suetsugu 2007). All family members are multi-domain proteins identified by sequence homology and binding interactions that serve to integrate signals for regulating WASp activity and sub-cellular localisation. The closest sequence homology is shared with N-WASp and common domains in these proteins have similar binding partners and functions. All WASp family proteins contain a characteristic C-terminal VCA domain (verprolin homology, central and acidic domain) capable of activating the actin-related protein (Arp)2/3 complex to initiate formation of new actin filaments. Cytosolic WASp adopts an autoinhibited conformation in which the VCA domain is associated with the proximal GTPase binding domain (GBD; Kim et al. 2000). Binding of the GTPase Cdc42 via a complex with Toca-1 results in disruption of the autoinhibited conformation, which releases the VCA domain and allows Arp2/3 and actin monomer binding (Buck et al. 2001; Ho et al. 2004; Lim et al. 2007). WASp-bound Arp2/3 complex is then able to mediate new actin polymerization, driving the assembly of a branched network of actin filaments and providing the mechanical propulsion for membrane protrusion, cell motility and cell shape changes (Millard et al. 2004).

Another mechanism for WASp activation is provided by phosphorylation, and has been described to occur in a variety of stimuli, including T cell receptor (TCR) stimulation, IgE receptor stimulation on mast cells and collagen receptor stimulation on platelets (Badour et al. 2004; Gross et al. 1999; Guinamard et al. 1998; Millard et al. 2004; Oda et al. 1998). An important residue for phosphorylation is tyrosine Y291, described as a target for Btk and Src family kinases (Badour et al. 2004; Cory et al. 2002; Guinamard et al. 1998; Torres and Rosen 2003, 2005). It is suggested that phosphorylation of tyrosine 291 lowers the threshold for Cdc42 activation and stabilises the ‘open’ molecular conformation of WASp (Torres and Rosen 2003, 2005), although there is also evidence that tyrosine 291 phosphorylation occurs independently of Cdc42 activation (Badour et al. 2004; Cory et al. 2002). Mutating tyrosine 291 to mimick its phosphorylated state renders WASp constitutively active and results in enhanced actin polymerization, demonstrating a direct effect of WASp phosphorylation on its function (Cory et al. 2002). All three constitutively active mutations found in patients with X-linked neutropenia are located in the GBD. They have been described to result in enhanced actin polymerization and dysregulated cell division, emphasising the importance of tightly regulated WASp activity (Ancliff et al. 2006; Devriendt et al. 2001; Moulding et al. 2007).

## Defective migration contributes to immunodeficiency

A hallmark of WAS is defective migration of immune cells, which is likely to be a major contributor to the observed immune dysfunction.

## Granulocytes

Granulocytes form the host's first line of defence against invading pathogens with neutrophils being amongst the first cells to respond. Migration of neutrophils is impaired in WAS KO mice both in vitro and in vivo (Snapper et al. 2005; Zhang et al. 2006), but human data are conflicting. While granulocytes isolated from patients with WAS demonstrated normal chemotaxis towards fMLP (Zicha et al. 1998), migration in response to c5a was shown to be impaired (Ochs et al. 1980). Defects in adhesion and migration of neutrophils in WAS appear to be subtle and more evident under conditions of physiologic shear flow, during which integrin attachment is critical, suggesting that ‘outside-in’ integrin signalling is defective in WASp deficiency (Zhang et al. 2006).

## Monocytes, macrophages and DCs

As with neutrophils, monocytes exit the circulation to enter inflamed tissues, where they can differentiate into macrophages or DC (Tacke and Randolph 2006). Monocytes, macrophages and DC from WAS patients are all defective in their ability to polarise and migrate in response to inflammatory chemokines in vitro (Altman et al. 1974; Badolato et al. 1998; Binks et al. 1998; Burns et al. 2001; Jones et al. 2002; Zicha et al. 1998). *In vivo* migration of murine DC is also impaired by WASp deficiency as shown by decreased migration of skin-resident DC and adoptively transferred DC to draining lymph nodes (Bouma et al. 2007; de Noronha et al. 2005; Snapper et al. 2005) and dyslocalisation of DC to the T cell areas of spleen or lymph nodes (Bouma et al. 2007; de Noronha et al. 2005). Contact of migrating myeloid cells with the substratum is thought to be mediated by podosomes, which are actin-rich structures that are surrounded by a ring of integrins and integrin-associated proteins. They are localised close to the leading edge of the cells and are important for cellular migration (Linder and Kopp 2005). WASp-deficient macrophages and DC display a striking lack of podosomes (Burns et al. 2001, 2004b; Calle et al. 2004; Jones et al. 2002; Linder et al. 1999). Down-regulation of WASp expression using lentiviral vector-mediated RNA interference induces identical morphological and migratory defects in DC from healthy individuals (Olivier et al. 2006), indicating a direct role for WASp in mediating the cytoskeletal defects that are observed in WASp-deficient cells. Restoration of WASp expression by gene replacement significantly restores podosome defects and improves the migratory ability in macrophages and DC both in vitro and in vivo (Blundell et al. 2008; Burns et al. 2001; Charrier et al. 2005; Jones et al. 2002).

## Lymphocytes

T and B lymphocytes of WAS patients and WAS KO mice have been shown to be impaired in their migratory capacity (Gallego et al. 2005; Snapper et al. 2005; Westerberg et al. 2005). While thymic T cell development appears to be relatively normal in WAS KO mice (Cotta-de-Almeida et al. 2007; Snapper et al. 1998; Zhang et al. 1999), reduced number of circulating T cells are found in WAS KO mice (Snapper et al. 1998) as well as WAS patients (Ochs et al. 1980; Park et al. 2004). However it remains to be investigated whether this is the resultant of reduced migratory potential or decreased survival. WASp-deficient B cells fail to migrate towards CXCL13 (Westerberg et al. 2005) and T cells do not respond to CCL19 and CCL21 (Snapper et al. 2005). Abnormal splenic architecture has been reported in both patients and mice, where both T cell areas and B cell follicles appear less well developed (Andreansky et al. 2005; de Noronha et al. 2005; Gerwin et al. 1996; Vermi et al. 1999; Westerberg et al. 2005). It is likely that impaired B and T cell migration is a contributing factor to the depleted white pulp of WAS spleen as the chemokine CXCL13 is thought to be crucial for attracting B cells to the lymphoid follicles (Ansel et al. 2000) and CCL19 and CCL21 mediate T cell homing to the T cell area of secondary lymphoid tissue (Gunn et al. 1999). Reduced homing potential of regulatory T cells, in addition to defective suppressor function, has recently been described, which could provide an explanation for the reduced number of peripheral regulatory T cells observed in WAS patients and WAS KO mice (Humblet-Baron et al. 2007; Maillard et al. 2007).

## Cell migration is important for initiating immune responses

**Fig. 1:**
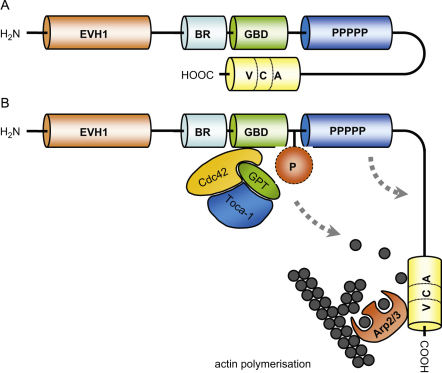
Schematic representation of regulation of WASp activity. Cytosolic WASp exists in an autoinhibited conformation in which the VCA domain is bound to the GBD (A). Activation of WASp by the Cdc42-GTP/Toca-1 complex or by phosphorylation of tyrosine 291 disrupts the autoinhibited conformation, enabling Arp2/3 binding and actin polymerization (B).

The efficacy of an immune response is dependent on both the spatial and temporal distribution of immune cells. Impaired migration of immune cells is likely to affect the mounting of an immune response in several aspects (Fig. 1). Firstly, impaired recruitment of neutrophils, monocytes and DC to inflammatory sites will allow longer time for pathogens to replicate and induce damage. Secondly, impaired migration of DC to draining lymphoid tissue will delay priming of antigen-specific T cells. This process is further hindered by defective uptake of particulate antigens by macrophages and DC. Thirdly, defective DC migration results in dyslocalisation of DC to the T cell area in lymphoid tissues such as spleen and lymph nodes, thus reducing the efficacy of encountering and activating antigen-specific T cells. And fourthly, defective migration of T cells means that homing of effector T cells to the inflammatory site will be reduced or delayed, further obstructing the ongoing immune response. It is clear that impaired migratory potential of immune cells is a significant contributor to dysregulated cellular localisation and a likely contributor to immunodeficiency.

## WASp deficiency affects immune cell effector function

In addition to a general defect of immune cell migration, effector function of most immune cells is affected by WASp deficiency.

## Cells of the innate immune system

**Fig. 2:**
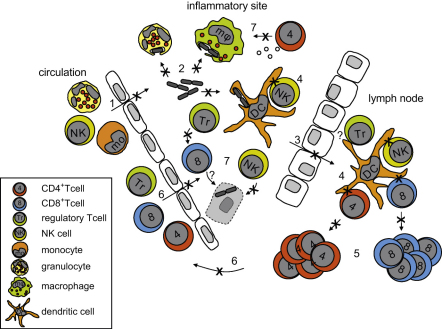
WASp deficiency impairs the mounting of the immune response. In response to invading pathogens, cells of the innate arm of the immune system, such as neutrophils and monocytes, will leave circulation and enter the inflamed tissue. Migration is impaired in WAS and this will likely result in defective extravasation of monocytes and neutrophils (1). Inside the tissue, pathogens will be phagocytosed by neutrophils, macrophages and DC (2), which is reduced in WASp-deficient cells. DC will migrate with the processed pathogen antigens to the draining lymph node (3), where they will present the antigens to lymphocytes (4). Both migration of DC and priming of T cells by DC is defective in WAS. Primed lymphocytes will proliferate (5), home to the inflamed tissue (6) and exert their effector function (7). WASp-deficient lymphocytes are impaired in proliferation and homing, and several effector functions are defective, such as suppressor function of regulatory T cells, lytic function of NK cells and cytokine production of CD4^+^ T cells. See text for references.

The ability to phagocytose is a key aspect of cells of the innate arm of the immune system. Phagocytosis is mediated by the formation of actin-based membrane invaginations, called phagocytic cups, that enclose extracellular particles and subsequently internalise these into so-called phagosomes (Swanson 2008). Monocytes of WAS patients are impaired in the uptake of FITC-labelled *Escherichia coli* (Lorenzi et al. 2000) and also macrophages show decreased phagocytosis of apoptotic cells and latex beads associated with defective phagocytic cup formation (Leverrier et al. 2001; Lorenzi et al. 2000) (Fig. 2). While granulocyte-mediated phagocytosis has not been investigated in WAS patients, this function is defective in WAS KO murine neutrophils (Zhang et al. 1999). WAS KO DCs have also been shown to be defective in their phagocytic capacity, although uptake of soluble antigens was normal (Westerberg et al. 2003). Knockdown of WASp by means of RNA interference confirmed the importance of WASp in mediating phagocytosis and phagocytic cup formation (Tsuboi and Meerloo 2007).

Only a few studies have been reported that investigate NK cell function in WAS. NK cells appear to have impaired lytic activity (Gismondi et al. 2004; Messina et al. 1986; Orange et al. 2002), which is most likely due to defective synapse formation with target cells (Orange et al. 2002) (Fig. 2).

## Cells of the adaptive immune system

Several cytoskeletal defects of WASp-deficient lymphocytes have been described, including reduced actin polymerization, abnormal morphology, cell polarisation and spreading and formation of membrane protrusions (Andreu et al. 2007; Facchetti et al. 1998; Molina et al. 1992, 1993; Westerberg et al. 2001, 2005). Proliferation in response to anti-IgM-mediated B cell receptor activation and in response to T cell receptor ligation are both impaired, while proliferation in response to mitogenic stimuli is unaffected (Gallego et al. 1997; Henriquez et al. 1994; Molina et al. 1993; Snapper et al. 1998; Zhang et al. 1999). Restoration of WASp expression by retroviral or lentiviral gene therapy has been shown to significantly improve T cell proliferation (Charrier et al. 2005, 2007; Dupre et al. 2004, 2006; Klein et al. 2003; Strom et al. 2003a, 2003b; Wada et al. 2002).

WASp-deficient CD4^+^ and CD8^+^ T cells fail to produce Th_1_ cytokines upon TCR activation (Fig. 2), which persists when T cells are cultured in Th_1_-polarising conditions (Morales-Tirado et al. 2004; Trifari et al. 2006). Interestingly, secretion of chemokines was normal (Morales-Tirado et al. 2004) and secretion of Th_2_ cytokines was only minimally affected (Trifari et al. 2006). It remains uncertain whether this is a specific defect for human WAS T cells, as in WAS KO mice, cytokine responses appeared normal (Nguyen et al. 2007). Memory T cell responses, however, were found to be impaired in WAS KO mice in response to influenza A virus (Andreansky et al. 2005; Strom et al. 2003b), although the role of priming by APC was not addressed in these studies.

Recently several reports have demonstrated impaired function of WASp-deficient regulatory T cells (Adriani et al. 2007; Humblet-Baron et al. 2007; Maillard et al. 2007; Marangoni et al. 2007). Although there does not seem to be consensus on whether regulatory T cell numbers in thymus and periphery are normal or reduced, it is clear that function of regulatory T cells both in vitro and in vivo is impaired.

There is a striking reduction in the number of marginal zone B cells in WAS KO mice (Meyer-Bahlburg et al. 2008; Westerberg et al. 2005, 2008) and of postgerminal CD27^+^ B cells, the human equivalent of marginal zone B cells, in WAS patients (Park et al. 2005), which is likely associated with the reduced antibody response to polysaccharide antigens (Ochs et al. 1980). Development of B cells appears normal with the exception of mature B cell subsets, in particular marginal zone B cells (Meyer-Bahlburg et al. 2008; Westerberg et al. 2008), although it is not clear whether impaired migration of B cell is a contributing factor in this defect.

## WASp in the immunological synapse

**Fig. 3:**
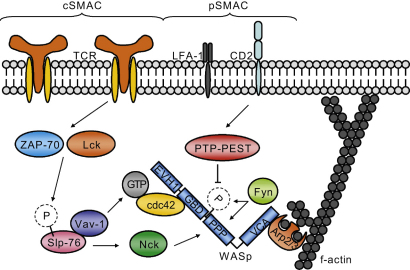
WASp recruitment and activation at the immunological synapse. TCR engagement initiates a signalling cascade involving the kinases ZAP-70 and Lck, which are involved in the phosphorylation of the adaptor molecule Slp-76. Phosphorylated Slp-76 interacts with WASp via Nck and recruits the GTPase Vav-1, which mediates activation of the Rho-GTPase Cdc42 and subsequently activates WASp. Another mechanism of WASp activation is proposed to be independent of Cdc42. The Fyn kinase binds to the polyproline domain (PPP) of WASp and phosphorylates tyrosine 291 to activate WASp. Regulation of this mechanism may be through dephosphorylation by PTP-PEST, which is recruited to the synapse by CD2. See text for references.

A major focus of research into the pathophysiology of WAS has been the role of WASp in TCR signalling. Formation of the immunological synapse at the interface between APC and T cells is required for optimal and appropriate T cell activation. Classically the IS is defined as a dynamic molecular structure formed around a central cluster of TCRs bound to peptide-loaded MHC molecules on the T cell and DC side, respectively. This central cluster is usually referred to as the central (c) supramolecular activation cluster (SMAC). LFA-1-ICAM-1 interactions and talin form a ring around the cSMAC and are referred to as peripheral (p) SMAC (Dustin et al. 2006; Monks et al. 1998). The IS ensures optimal T cell activation, although T cell activation can also occur in the absence of cSMAC formation (Lee et al. 2003). The cSMAC has been implicated as a site for receptor degradation (Varma et al. 2006), indicating that in order for the IS to be maintained, continuous signalling and recruitment of molecules is required. WASp-deficient T cells display a well-documented defect of proliferation and IL-2 production in response to TCR stimulation (Cannon and Burkhardt 2004; Dupre et al. 2002; Gallego et al. 1997; Molina et al. 1993; Snapper et al. 1998; Zhang et al. 1999) (Fig. 2). Directly after TCR stimulation WASp is recruited to lipid rafts (Dupre et al. 2002), which are required for IS formation (Dykstra et al. 2003). In the absence of WASp, T cells failed to aggregate lipid rafts to the synapse site (Dupre et al. 2002). Targeting WASp to the IS is likely to be mediated by its polyproline domain, as a mutated WASp in which the polyproline domain was deleted failed to localise to the IS, in contrast to a GBD-deleted WASp mutant (Badour et al. 2003; Cannon et al. 2001). Several adaptor molecules have been implicated in mediating WASp activation and recruitment to the IS (Fig. 3). TCR signalling induces accumulation of ZAP-70 to lipid rafts and subsequently to the IS (Sasahara et al. 2002). ZAP-70 and Lck recruitment has been shown to be instrumental for the accumulation of Cdc42-GTP to the synapse and leads to phosphorylation of the adaptor molecule Slp-76 (Zeng et al. 2003). Slp-76 has been proposed to recruit WASp through binding to the SH-3 domain of Nck on one hand, while on the other hand it recruits the GTPase Vav-1, which mediates activation of Cdc42 and subsequently activation of WASp (Zeng et al. 2003). Although Cdc42 is an important activator of WASp, its role may be redundant in WASp activation at the IS (Cannon et al. 2001). Activation of WASp at the IS has also been shown to be mediated by the Fyn kinase, which binds the polyproline domain of WASp via its SH3 domain and phosphorylates tyrosine 291 (Fig. 3) (Badour et al. 2004). This activational mechanism was independent of Cdc42-mediated WASp activation and potentially regulated by dephosphorylation of Y291 by PTP-PEST, which interacts with WASp via binding to PSTPIP1 and could be recruited to the synapse by CD2 (Badour et al. 2003, 2004). A further role for WASp has been implicated in regulating dynamics of the IS by Sims et al. (2007), who showed that although the IS was initially formed in WASp-deficient T cells, symmetry of the pSMAC was lacking, resulting in disruption and failure of the IS to re-form.

Most of these studies have been performed using B cell–T cell conjugates, but interestingly when EL4 thymoma cells or lipid bilayers were used as antigen-presenting cells no defects were observed in the ability of the WASp null T cells to form conjugates (Krawczyk et al. 2002; Sims et al. 2007). It has been suggested that defective synapse formation in WASp-deficient T cells is dependent on antigen dose (Cannon and Burkhardt 2004) and differences in MHC:TCR affinity could explain why normal conjugates were formed when using AND, P14 or DO11.10 TCR transgenic WASp null T cells, but not when using OTII TCR transgenic WASp null cells (Badour et al. 2003, 2004; Cannon et al. 2001; Cannon and Burkhardt 2004; Krawczyk et al. 2002; Sims et al. 2007).

## DC regulation of immunological synapse formation

Recent findings have indicated that interaction of WAS KO DC with normal T cells resulted in reduced antigen-specific CD4^+^ and CD8^+^ T lymphocyte proliferation (Bouma et al. 2007; Pulecio et al. 2008). Although impaired migration of DC contributes to reduced T cell responses, the direct interaction between DC and T cells is also hampered (Fig. 2). *In vitro* studies, which eliminate migration as confounder, have shown that priming of WASp-sufficient T cells by antigen-loaded WASp-deficient DC is indeed reduced (Bouma et al. 2007) and correcting the reduced number of DC that reach the lymph nodes in vivo did not rescue defective T cell priming by WAS KO DC (Pulecio et al. 2008). In addition, WASp-expressing T cells failed to form stable contacts with WASp null DC either in vitro or in vivo (Pulecio et al. 2008), suggesting that DC-mediated IS formation could be defective. A similar defect has been reported previously, when it was shown that WASp null DC fail to form an immunostimulatory synapse with wild-type NK cells (Borg et al. 2004). These data suggest that WASp-deficient DC will fail to form a stable, functional IS with T cells, although this remains to be formally investigated. There is ample evidence that IS formation is impaired in WASp-deficient T cells (Badour et al. 2003, 2004; Cannon et al. 2001; Cannon and Burkhardt 2004; Dupre et al. 2002; Sasahara et al. 2002; Zeng et al. 2003), and it is highly likely that when both DC and T cells lack WASp, the quality and function of the IS will be further reduced. Indeed, an important role for the APC in the formation of the IS is suggested by the findings that defects in IS formation could be rescued by modulating affinity of TCR–MHC interactions through antigen dose or varying choice of APC or TCR (Badour et al. 2003, 2004; Cannon et al. 2001; Cannon and Burkhardt 2004; Krawczyk et al. 2002; Sims et al. 2007). Recently, it was reported that WASp-deficient B cells fail to accumulate LFA-1 molecules to the pSMAC and fail to form a mature IS (Meyer-Bahlburg et al. 2008), suggesting that in addition to DC, B cell APC function may be deficient as well. Given the defective suppressor function of regulatory T cells in WAS, it would be very interesting to investigate whether defective regulatory T cell function is the result of improper APC activation, as was shown for WASp-deficient DC activation of CD4^+^ and CD8^+^ T cells (Bouma et al. 2007; Pulecio et al. 2008).

## Concluding remarks

Taken together there is compelling evidence that migration of most immune cell lineages is defective as a result of lack of WASp expression. This most likely has a significant impact on the efficacy of immune cells to reach sites of infection and to transport antigens to draining secondary lymphoid tissue. Furthermore, activation of T cells is impaired. This is probably due to reduced proliferative capacity of the T cells themselves on the one hand and to defective IS formation on the other hand. It is becoming clear that WASp is involved not only at the T cell side of the IS but also in the APC. We would like to speculate that WASp deficiency in the APC is an important factor in defective IS formation, resulting in defective T cell education, but this needs further investigation. Taken together, reduced migration and defective activation of immune cells will impair, or at least delay, the mounting of an immune response, resulting in compromised immunity.
